# Whole Exome Sequencing Aids the Diagnosis of Fetal Skeletal Dysplasia

**DOI:** 10.3389/fgene.2021.599863

**Published:** 2021-03-10

**Authors:** Hui Tang, Qin Zhang, Jingjing Xiang, Linliang Yin, Jing Wang, Ting Wang

**Affiliations:** ^1^Center for Reproduction and Genetics, The Affiliated Suzhou Hospital of Nanjing Medical University, Suzhou, China; ^2^Center for Reproduction and Genetics, Suzhou Municipal Hospital, Suzhou, China; ^3^Suzhou Guangji Hospital, Suzhou, China

**Keywords:** skeletal dysplasia, prenatal diagnosis, whole-exome sequencing, SNP-array, novel variants

## Abstract

Skeletal dysplasia is a complex group of bone and cartilage disorders with strong clinical and genetic heterogeneity. Several types have prenatal phenotypes, and it is difficult to make a molecular diagnosis rapidly. In this study, the genetic cause of 16 Chinese fetuses with skeletal dysplasia were analyzed, and 12 cases yielded positive results including one deletion in *DMD* gene detected by SNP-array and 14 variants in other 6 genes detected by whole exome sequencing (WES). In addition, somatic mosaicism was observed. Our study expanded the pathogenic variant spectrum and elucidated the utilization of WES in improving the diagnosis yield of skeletal dysplasia.

## Introduction

Unexpected skeletal dysplasia affects approximately 1 per 5,000 and is a complex group of bone and cartilage disorders with strong clinical and genetic heterogeneity (Geister and Camper, 2015). In the 2015 revision of the Nosology and Classification of Genetic Skeletal Disorders, 436 disorders are classified into 42 groups according to syndromes, publication, genetic information, and nosologic autonomy, and 364 different genes are associated with genetic skeletal disorders (Bonafe et al., 2015). Many of these disorders can give rise to prenatal phenotypes. In the past, ultrasound evaluation was a widely used method for detection of congenital anomalies (Krakow et al., 2009). However, the lack of family history and non-specific and limited clinical symptoms *in utero* may introduce difficulties in prenatal diagnosis. Recently, with the advances in molecular technology, especially next-generation sequencing, high-throughput sequencing has been considered as an effective method for different genetic diagnosis (Retterer et al., 2016; LaDuca et al., 2017). Based on the statements released by the International Society for Prenatal Diagnosis (ISPD), the Society for Maternal Fetal Medicine (SMFM), and the Perinatal Quality Foundation (PQF), and the American College of Medical Genetics and Genomics (ACMG), next-generation sequencing can be used with ultrasound anomalies when standard diagnostic genetic testing, such as chromosomal microarray analysis, failed to yield a definitive diagnosis. Especially, if a specific diagnosis is suspected, molecular testing for the suggested disorder should be the initial test. Thus, many studies have adopted this for prenatal evaluation (International Society for Prenatal et al., 2018; Monaghan et al., 2020). Thirty-one studies conducted prenatal analysis by whole exome sequencing (WES) with the diagnostic rates between 6.2 and 80% (Best et al., 2018). Notably, the application of targeted exome sequencing in prenatal diagnosis of skeletal dysplasia is outstanding as several researches have reported high detection rates from 75 to 83.3% (Chandler et al., 2018; Zhou et al., 2018; Liu et al., 2019; Han et al., 2020). Definitive molecular diagnosis can provide accurate results instead of a suspected clinical impression and information about subsequent development of the disease and treatment regimens; thus, parents could get genetic counseling, and birth-defect intervention could be implemented for future pregnancies.

In this study, we analyzed 16 cases of fetuses with suspected skeletal dysplasia by WES and aimed to elucidate WES as a useful and efficient aid to precise diagnosis.

## Materials and Methods

### Patients

This study was approved by the institutional ethics committee of the Affiliated Suzhou Hospital of Nanjing Medical University. Sixteen affected patients and available family members were recruited with informed consent. All fetuses were diagnosed with suspected skeletal abnormalities by prenatal ultrasound and some ultrasound pictures were listed in **Figure 2**. Their clinical symptoms are summarized in Table 1. We obtained fetal muscle tissue or cord blood and parents' peripheral blood with the exception of cases 9 and 10 that we only got the samples from the patients. Genomic DNA was extracted using the QIAamp DNA Blood Mini Kit (Qiagen, Hilden, Germany) according to standard extraction methods.

**Table 1:** Summary of clinical findings and molecular diagnoses.

**Case**	**Family history**	**Gestation (w)**	**Ultrasound findings**		**Molecular result**		**Variant Type** **(ACMG)**	**MAF** **(gnomAD)**	**References or** ***in silico* prediction**
			**Skeletal anomalies**	**Other anomalies**	**Variant**	**Inheritance**			
1	One previous pregnancies with the same anomalies	24	Feet were ballet-shaped, and the angle of tibiofibula and dorsum of foot was near 180°	Polyhydramnios	arr[hg19] Xp21.1(31,690,978-31,875,673)x0	Maternal	Pathogenic (PVS1, PS4, PM2)	-	Murugan et al., 2010
2	Previous pregnancy with short long bones and narrow thorax	25	Narrow thorax TH = 118 mm, short long bones FL = 23 mm HL = 19 mm, polydactylia	Renalsinusseparation	*DYNC2H1* NM_001080463 c.5984C>T and c.10606C>T	AR; Biparental inheritance	Pathogenic (PS4, PM2, PM3, PP4)and Pathogenic (PS4, PM2, PM3, PP4)	0.000004030 (1/248124); 0.00003 (1/31386)	(c.5984C>T and c.10606C>T: Qiao et al., 2018)
3		26	Short and curved long bones, FL = 34 mm HL = 36 mm, curved cubitus		*ALPL* NM_000478.5 c.984_986del and c.1463C>G	AR; Biparental inheritance	Pathogenic (PS4, PS3, PM4) and Likely Pathogenic (PM1, PM2, PM3)	0.00001062 (3/282562); -	(c.984_986del : Chang et al., 2012; Taillandier et al., 2015; c.1463C>G: 0.9885, P, B, B)
4		22	Short and curved long bones	Cloverleaf skull, enteric canal echo enhancement	*ALPL* NM_000478.5 c.984_986del and c.2T>C	AR; Biparental inheritance	Pathogenic (PS4, PS3, PM4) and Pathogenic (PVS1, PM2, PM3)	0.000008 (2/251166); 0.000004 (1/251344)	(c.984_986del : Chang et al., 2012; Taillandier et al., 2015; c.2T>C: 0.9808, P, P, P)
5		25	Narrow thorax, short long bones, FL = 24 mm HL = 17.6 mm, short rib		*FGFR3* NM_000142.4 c.742C>T	*De novo*	Pathogenic (PS2, PS4)	-	Pokharel et al., 1996; Sawai et al., 1999; Chen et al., 2001; Tonni et al., 2010
6		24^+6^	Short long bones, FL = 29.4 mm HL = 17 mm		*COL1A2* NM_000089 c.2189G>T	*De novo*	Pathogenic (PS2, PS1, PM1, PM2)	-	0.9871, P, P, P
7		14^+3^	X-type lower limbs, upper limbs adductus,nasalbonelength 2.2 mm	NT = 4 mm anasarca					
8	Two previous pregnancies with the same anomalies	19	Short long bones with fracture, short rib, wide orbilal septum		*COL1A2* NM_000089 c.1764+3_1764+6del	Maternal	VOUS (PM2, PP1, PP3)	-	-, P, -, -
9		25	Short long bones, lumbosacral portion bent						
10		24	Short limbs, micrognathia, spine misaligned		*HSPG2* NM_005529.6 c.8553del and c.12532+1G>T	U	Likely Pathogenic (PVS1, PM2) and Pathogenic (PVS1, PM2, PM3)	-; 0.00000401 (2/249388)	c.8553del: -, P, -, -; c.12532+1G>T: 0.9958, P, -, -
11		31	Abnormal morphology of ulna						
12	Two previous pregnancies with the same anomalies	23	Short limbs, narrow thorax, bell-shaped chest	Anasarca CTR>0.5 pleural effusion	*COL1A1* NM_000088.3 c.3389G>A	Maternal	Likely Pathogenic (PM1, PM2, PP3, PP5)	-	ClinVar
13		20	Short long bones with abnormal thorax	NT = 4.4 mm	*COL1A1* NM_000088.3 c.1921G>A	*De novo*	Pathogenic (PS2, PM1, PM2, PM5)	-	0.9981, P, P, P
14		25	Short and curved long bones		*COL1A2* NM_000089 c.1010G>A	*De novo*	Pathogenic (PS2, PM1, PM2, PM5)	-	0.9974, P, P, P
15		22	Short left humerus and curved ulnaandradius						
16		U	Short long bones		*FGFR3* NM_000142.4c.1138G>A	*De novo*	Pathogenic (PS2, PS4)	-	Xue et al., 2014

*TH, Thoracic Circumference; FL, Femur length; HL, humerus length; NT, nuchal translucency; CTR,cardiothoracic ration; AD, autosomal dominant; AR, autosomal recessive; U, unknown; Four prediction tools: DANN, MutationTaster, REVEL and SIFT:-, unpredictable; P, Pathogenic; B, Benign. The value range of DANN is 0 to 1, with 1 given to the variants predicted to be the most damaging*.

### SNP-array

Single nucleotide polymorphism array analysis was performed on the Affymetrix CytoScan platform (Affymetrix, Santa Clara, CA, USA) following the protocol. After amplified, DNA was hybridized to the Affymetrix 750K array containing 550,000 copy number variation (CNV) markers and 200,000 SNP markers. Data were analyzed by Chromosome Analysis Suite 3.2 (Affymetrix, Santa Clara, CA, USA). CNVs were analyzed and classified according to the guidelines released by American College of Medical Genetics and Genomics (Kearney et al., 2011).

### Whole-Exome Sequencing and Mutation Analysis

WES was performed by the Fulgent Genetics Company (Fuzhou, China). DNA of the fetus and their parents were used to create the DNA libraries, enriched by IDT xGen Exome V2 reagent (Integrated DNA Technologies, Inc., Iowa, United States), sequenced on the Illumina Nova6000 (Illumina, San Diego, CA, United States), and mapped to the human genome (NCBI37/hg19) by the Sentieon software package. Over 99% coverage of targeted bases was achieved, with an average sequencing depth of over 100. Then Picard was used to compare the results to remove redundancy, and the variants were detected by vVarscan. An in-house algorithm CNVexon developed by Fulgent Genetics was used for exon-based CNV detection based on reads counts, and a misalignment detection algorithm was used for pseudogenes' optimization. All variants were searched in multiple databases including gnomAD, Exome Aggregation Consortium (ExAC), dbSNP, 1000 Genome Project, Human Gene Mutation Database (HGMD), ClinVar and Leiden Open Variation Database (LOVD). Four prediction tools were used for variant interpretation: DANN, MutationTaster, REVEL, and SIFT. For splicing variants, Human Splicing Finder was used. Variants were classified according to the recommended guidelines released by the American College of Medical Genetics and Genomics and the Association for Molecular Pathology (Richards et al., 2015).

### Verification of Gene Mutations

If microdeletions or microduplications were detected, these would be verified by multiplex ligation-dependent probe amplification (MLPA) in patients and parents. And if mutations were detected for a single gene, Sanger sequencing would be conducted to validate the mutations.

## Results

A total of 16 cases with suspected skeletal dysplasia were investigated, and all of them underwent detailed ultrasound examination during pregnancy. Several parameters were examined by ultrasound: biparietal diameter, head circumference, abdominal circumference, chest circumference, length of the long bones, shape of long bones, mandibular size and shape, abnormal posturing of the extremities, and other congenital anomalies. In 16 cases, we detected one deletion in *DMD* and causative variants in six genes, including *FGFR3, COL1A1, COL1A2, ALPL, HSPG2*, and *DYNC2H1* with a detection rate of 75% (Table 1).

### Abnormalities Detected by SNP-array

**Figure 1:**
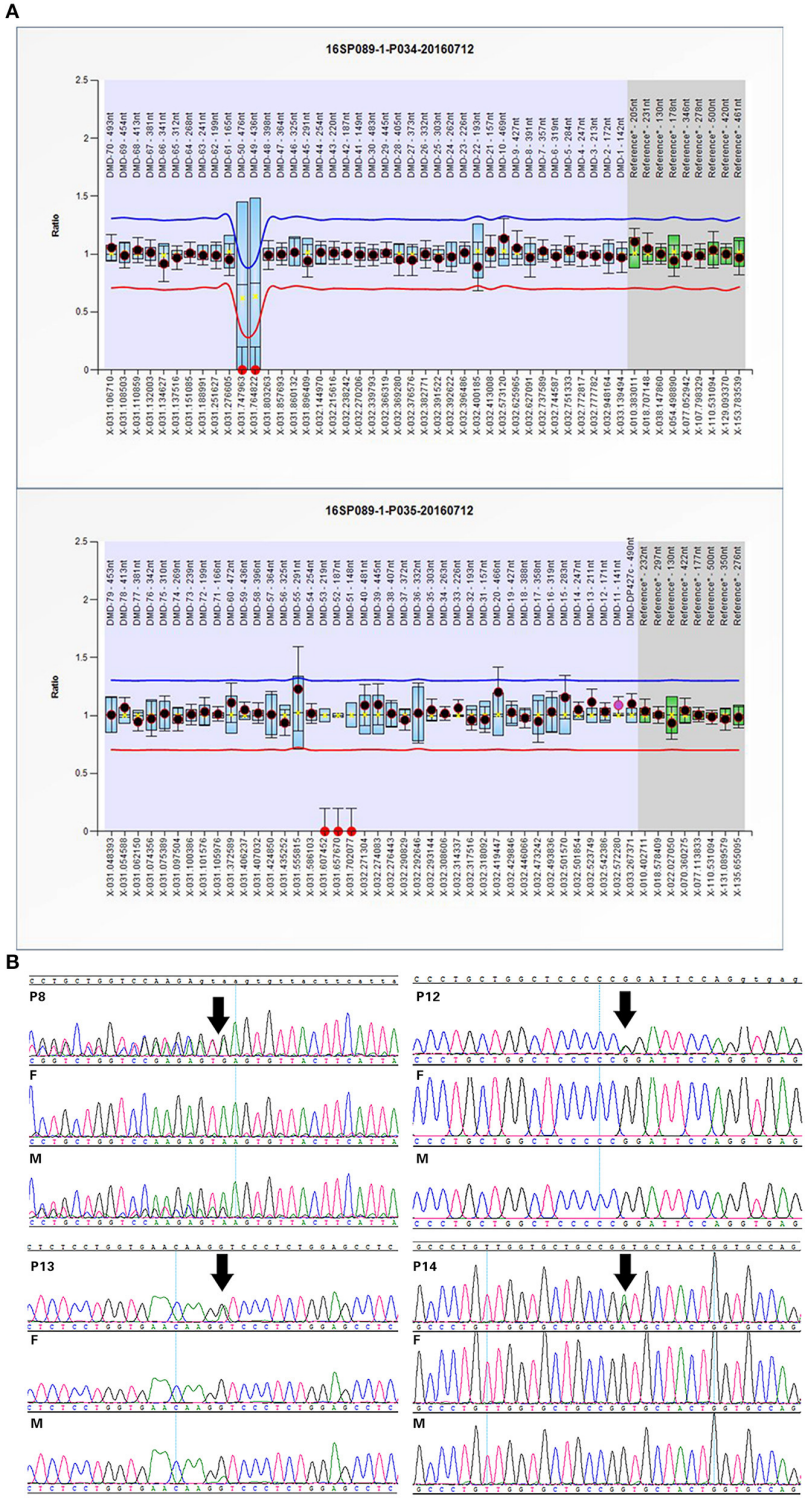
Confirmation of SNP-array or WES results: **(A)** The MLPA results of case 1 indicated the deletion spanning from exon 49 to exon 53 in *DMD* gene. **(B)** Sanger sequencing confirmed mutations in some cases with OI. Variants are indicated by black arrows.

Twelve cases were tested for CNVs first by SNP-array. We found a 184 kb deletion in chromosome Xp21.1, which spanned from exon 49 to exon 53 of the *DMD* gene in case 1. At 24 weeks of gestation, the ultrasound scan showed that the fetal feet were ballet-shaped, and the angle of tibiofibula and dorsum of foot was near 180°. However, the fetus didn't receive a prenatal genetic test. When he was born, an SNP-array was conducted using his cord blood. Further analysis by MLPA verified the deletion in the patient, and his mother was heterozygous for this deletion (Figure 1A).

### Abnormalities Detected by WES

WES was performed for 11 cases with negative results of SNP-array analysis and other four new cases. A total of 14 variants in 6 genes associated with skeletal dysplasia were detected in 11 cases (Table 1) and confirmed by Sanger sequencing (Figure 1B).

In case 2, two compound heterozygous causative mutations c.5984C>T (p.A1995V) and c.10606C>T (p.R3536X) (Qiao et al., 2018) in *DYNC2H1* gene were detected. Mutations in *DYNC2H1* were associated with short-rib thoracic dysplasia 3 (Merrill et al., 2009). Short rib-polydactyly syndrome 3 (SRPS 3) was an autosomal recessive disease overlapping with Jeune asphyxiating thoracic dystrophy belonging to the ciliopathy, but it was more severe and characterized by early prenatal expression, lethality and variable malformations (Dagoneau et al., 2009), which was correlated with patient 2′s presentations.

Hypophosphatasia resulting from mutations in *ALPL* gene was found in case 3 (c.984_986del, c.1463C>G) and case 4 (c.2T>C, c.984_986del), respectively. Both of the mutations in these two cases presented compound heterozygous condition. c.984_986del leaded to the deletion of phenylalanine at position 328 at the β-sheet. c.2T>C caused the loss of the start codon, and the alanine residue at position 488 (c.1463C>G, p.A488G) was conserved in several species.

Mutations c.742C>T was detected in case 5, and c.1138G>A was detected in case 16, which were mutation hotspots of *FGFR3* gene. Mutations in collagen genes were identified in five cases, of which c.2189G>T (p.G730V) in *COL1A2* of case 6, c.1921G>A (p.G641R) in *COL1A1* of case 13, and c.1010G>A (p.G337D) in *COL1A2* of case 14 were *de novo*, while c.1764+3_1764+6delAAGT in *COL1A2* of case 8 and c.3389G>A in *COL1A1* of case 12 were maternally inherited. Four missense mutations resulted in glycine substitutions in Gly-X-Y, which could create severe damage to collagen. The splicing mutation c.1764+3_1764+6delAAGT was predicted to disrupt normal splicing by Human Splicing Finder.

In case 10, clinically significant mutations in *HSPG2* were found (c.8553del and c.12532+1G>T). c.8553del produced a truncated protein that terminated at 2878 amino acid residues lacking part of domain IV and the whole domainV, and c.12532+1G>T at the C-terminal region may lead to abnormal splicing as predicted by Human Splicing Finder. However, no pathogenic variants were identified in cases 7, 9, 11, and 15.

## Discussion

Many aspects could affect the detection rate of WES, such as the number of cases, selected criteria of study, proband-only WES, or trio WES, and so on (Best et al., 2018). It was reported that trio WES had a higher diagnostic rate; fetuses with multiple anomalies also had a higher diagnostic rate and when testing single structural anomalies, a particular organ system may have a higher diagnostic yield (Best et al., 2018; International Society for Prenatal et al., 2018). Among the 16 cases in the present study, 12 cases received a definitive molecular diagnosis, including a microdeletion and 8 novel variants, and the detection rate is 75%, which is consistent with the high detection rates of skeletal dysplasia from 75 to 83.3% revealed by previous studies (Chandler et al., 2018; Zhou et al., 2018; Liu et al., 2019; Han et al., 2020).

In case 1, a deletion spanning from exon 49 to exon 53 of the *DMD* gene was detected in a male patient and inherited from his mother. He exhibited abnormal posturing of the lower extremities, which were not reported previously during the intrauterine period in patients with DMD/BMD. And the family has experienced one pregnancy with the same anomalies before without other information and further genetic analysis. To our knowledge, only one fetus with a deletion of exons 17–29 of the *DMD* gene was reported to present prenatal phenotypes including fetal growth restriction and oligohydramnios (Lin et al., 2017). And the deletion of exons 49–53 of the *DMD* gene were detected in three patients with DMD/BMD as reported previously (Covone et al., 1991; Murugan et al., 2010; Yang et al., 2019). However, our patient was dead, and we could not get more information. Therefore, it is difficult to define these new symptoms in intrauterine period as phenotype expansion of DMD/BMD or genotype-phenotype discordance.

Moreover, we found mutations in *ALPL* in cases 3 and 4. Pathogenic variants in *ALPL* cause hypophosphatasia characterized by defective mineralization of bone and/or teeth in the presence of low activity of serum and bone alkaline phosphatase (ALP) (Millan and Whyte, 2016). c.984_986del in cases 3 and 4 was reported previously (Chang et al., 2012; Taillandier et al., 2015), which result in the deletion of phenylalanine at the β-sheet and decreased activity of its coding protein the tissue-nonspecific isoenzymes of alkaline phosphatase (TNSALP) (Michigami et al., 2005). Thus, c.984_986del may reduce the enzymatic activity too. c.1460C>T (p.A487V) and c.1466G>C (p.C489S) were reported early, of which the latter one exhibited a diminished ALP activity, less located on the cell surface and failed to become the mature form (Satou et al., 2012; Porntaveetus et al., 2017). This suggested that p.487-489 of ALPL protein may play an important role in enzymatic activity. c.2T>C caused the loss of the start codon and generated a transcript starting at Met56 as c.3G>A did, which did not exhibit enzymatic activity, had no significant effect on the wild type ALPL protein, and could not be attached to the cell membrane (Mentrup et al., 2011).

Patient 5 harbored c.742C>T in *FGFR3* gene, which has been detected in different populations (Pokharel et al., 1996; Sawai et al., 1999; Chen et al., 2001; Tonni et al., 2010). And patient 16 had a hotspot mutation c.1138G>A in *FGFR3* (Xue et al., 2014). These fetuses' ultrasound scans all revealed a narrow chest with shortening of the long bones. Besides, c.8553del and c.12532+1G>T in *HSPG2* were identified in case 10. *HSPG2* is an essential gene, and its mutations could lead to Schwartz-Jampel syndrome, type 1(SJS) and severe neonatal lethal Dyssegmental dysplasia, Silverman-Handmaker type (DDSH) (Arikawa-Hirasawa et al., 2002; Martinez et al., 2018). However, the compound heterozygous condition couldn't be confirmed because the parents' samples were not available. Trio sequencing of patient 7 and the parents detected a heterozygous missense variation c.4813C>T in *FLNB*, which was inherited from the normal father with 62 of 120 (51.7%) reads. Though several cases with family history presenting an autosomal dominant trait has been reported (Doren et al., 1998; Xu et al., 2018), the father in case 7 didn't have malformations associated with FLNB-related disorders such as short stature, club feet, and facial dysmorphisms (Bicknell et al., 2007).

**Figure 2:**
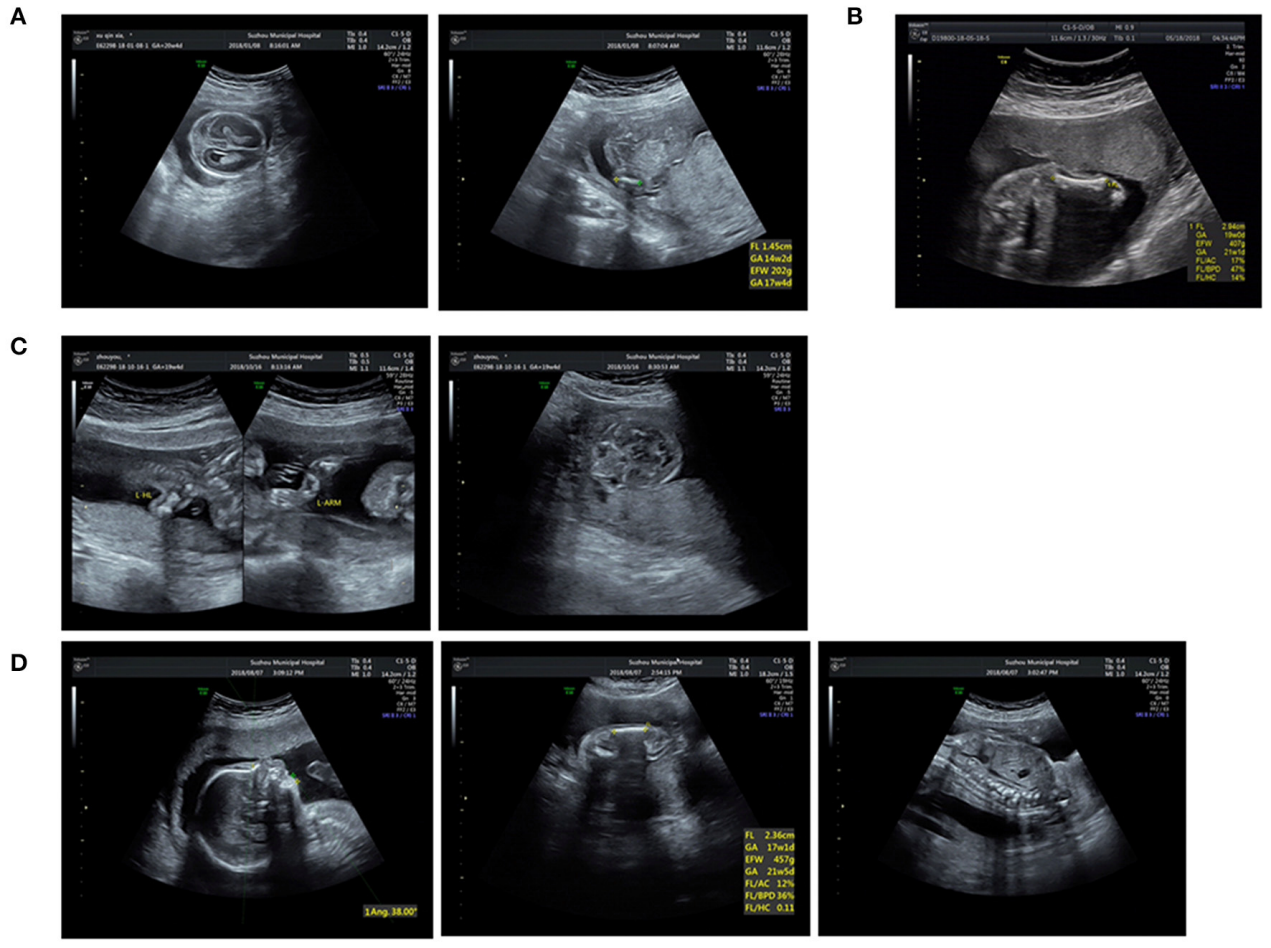
Ultrasound pictures of cases 4, 6, 8, and 10: **(A)** Short femur and cloverleaf skull of P4. **(B)** Short femur of P6. **(C)** Short long bones and wide orbilal septum of P8. **(D)** Short limbs, micrognathia, and spine misaligned of P10.

Furthermore, variants of type I collagen genes related to osteogenesis imperfect (OI) were identified in five cases. Type I collagen is a heterotrimer containing two α1(I) and one α2(I) chains assembled by procollagen chains with N-terminal and C-terminal globular propeptides flanking the helical domain (Forlino and Marini, 2016). The helical domains contain Gly-X-Y triplets where glycine substitutions are the most frequent cause of OI (Marini et al., 2007). In our study, we detected four missense mutations and one splicing variant: c.1921G>A and c.3389G>A in *COL1A1* and c.1010G>A, c.2189G>T, and c.1764+3_1764+6del in *COL1A2*, respectively (Figure 2B). Four missense mutations were glycine substitutions in Gly-X-Y, which would delay helical folding and prolong access time for modifying enzymes. Previous researches have described two infants with c.2188G>T (p.G730C) and c.2188G>C (p.G730R) in *COL1A2* changing the same amino acid residue as c.2189G>T (p.G730V) and exhibited the same phenotypes such as blue sclera, wormian bones, and shortening and bowing of the upper and lower limbs (Gomez-Lira et al., 1994; Hachiya et al., 2012). The similar situation was observed in c.1010G>A and c.1921G>A. And c.3389G>A in *COL1A1* has been classified as likely pathogenic in ClinVar (https://www.ncbi.nlm.nih.gov/clinvar/). The splicing mutation c.1764+3_1764+6del in *COL1A2* in case 8 may yield abnormal splicing transcript as predicted by Human Splicing Finder. Notably, the variants in cases 8 and 12 were maternally inherited. Both of the mothers experienced induced abortion twice in the second trimester due to the same skeletal dysplasia malformation, suggesting the somatic mosaicism. The mutated allele c.3389G>A was present in 17 of 66 (25.8%) reads in WES, and the peak of mutated allele was lower than that of the wild-type allele in sanger sequencing, suggesting that it is a mosaic mutation in the mother of case 12. After that, we tried to recall the asymptomatic mother; however, she didn't receive radiographical examination, and we only knew that her sclera and height were normal and didn't suffer bone fracture before. Similarly, the mutated allele c.1764+3_1764+6del was present in 22 of 68 (32.3%) reads in the mother of case 8 who presented extremely mild symptoms such as short stature compared with the fetus. Nevertheless, the ratio of the fetus was 37.8% (17/45) similar to the mother. We supposed that other factors such as underlying genetic modifiers may affect the phenotypes.

Four cases did not have a definitive molecular diagnosis, and the negative WES results could be attributed to limited phenotypes or the limitations of WES. For fetuses, several aspects could affect the detection rate, especially incomplete prenatal phenotypes (Aarabi et al., 2018). Meanwhile, many challenges should be considered, such as ethical concerns, analysis of variants of unknown significance, and secondary findings (Best et al., 2018; Monaghan et al., 2020). In the future, with the development of fetal specific phenotype genotype database, the uncertainty in cases will become less frequent (Aarabi et al., 2018). Furthermore, the causative variants of these four cases may reside in the noncoding-regulatory or deep-intronic regions could not be detected by WES, which could be analyzed by whole genome sequencing in the future.

## Conclusion

In summary, one CNV and 14 single nucleotide variants of 6 genes were identified in 16 families with suspected skeletal abnormalities by prenatal ultrasound scan. The results of this study elucidated that the utilization of WES improved the diagnosis yield of skeletal dysplasia and provided useful genetic counseling guidance for parents. In addition, two cases with type I collagen variants from asymptomatic parent were also found, indicating the advantage of next generation sequencing in the detection of somatic mosaicism. Further studies will be needed to evaluate the application of prenatal WES for skeletal dysplasia.

## Data Availability Statement

The datasets for this article are not publicly available due to concerns regarding participant/patient anonymity. Requests to access the datasets should be directed to the corresponding author.

## Ethics Statement

The studies involving human participants were reviewed and approved by The Affiliated Suzhou Hospital of Nanjing Medical University. Written informed consent to participate in this study was provided by the participants' legal guardian/next of kin. Written informed consent was obtained from the minor(s)' legal guardian/next of kin for the publication of any potentially identifiable images or data included in this article.

## Author Contributions

TW and JW were responsible for testing strategy design. HT and JX analyzed the data and drafted the manuscript. QZ and LY provided clinical information. HT and JX carried out the molecular analyses. All authors read and approved the final manuscript.

## Conflict of Interest

The authors declare that the research was conducted in the absence of any commercial or financial relationships that could be construed as a potential conflict of interest.
